# Structure–Properties Relationships Involved in the Embrittlement of Epoxies

**DOI:** 10.3390/polym14214685

**Published:** 2022-11-02

**Authors:** Romain Delannoy, Vincent Tognetti, Emmanuel Richaud

**Affiliations:** 1 Laboratoire PIMM, Arts et Metiers Institute of Technology, CNRS, Cnam, HESAM Universite, 151 Boulevard de l’Hopital, 75013 Paris, France; 2 Normandy Univ., Cobra Umr 6014 & FR 3038, Université de Rouen, INSA Rouen, CNRS, 1 rue Tesniere, CEDEX 09, 76821 Mont St. Aignan, France

**Keywords:** epoxy-diamine networks, thermal oxidation, embrittlement

## Abstract

This paper illustrates a study of the thermal oxidation of several epoxy amine networks. Oxidation was followed at the molecular scale using Fourier Transform InfraRed spectroscopy (FTIR) and at the macromolecular scale using tensile tests. FTIR showed the major formation of amides, while tensile tests showed the progressive increase in the elastic modulus (~0.5 GPa for room temperature Young modulus) and decrease in ultimate strain and volumic energy for failure (assessed using the integrals of stress-strain curves). Both ultimate strain and volumic energy were divided by more than two. Linear correlations between oxidation trackers (amide concentration) and changes in mechanical parameters are presented and discussed.

## 1. Introduction

Epoxy-amine networks are easily obtained from the condensation reaction between polyglycidyl molecules and amines. Since there is a quasi “infinity” of networks differing by the flexibility of monomers and crosslink densities, epoxy-amine networks are thus undoubtedly the most investigated family of thermoset polymers in terms of structure–properties relationships [1,2,3,4]. From an industrial point of view, they are used in demanding applications where they are submitted to enhanced temperatures and the presence of chemicals (oxygen, or water, for example) likely to induce their degradation [5,6]. Ageing in the presence of water is in principle reversible contrary to chemical ageing by thermal oxidation where changes in chemical and macromolecular structure are irreversible and lead to the definitive failure of material.

There is already an exhaustive literature on the nature of the chemical trackers of the oxidation of epoxies [7,8,9,10]. Mechanisms responsible for their formation are in greater part understood and already derived in sophisticated kinetics models predicting the changes in chemical structure [11]. This fulfils, in part, the need for predicting lifetime; however, there is still a scientific gap to bridge for converting those structural modification into mechanical changes so as to evaluate the time at which mechanical properties fall below a critical level out of the end-user specifications. The causal chain has already been investigated. Oxidation induces chain scissions and/or crosslinking (depending on the flexibility of the segments between crosslinks) and mass loss. This results in glass transition temperature (T_g_) changes and « antiplasticization » induced by the destruction of small groups (α-hydroxypropylether) and the loss of sub-glassy mobility [12,13]. This last phenomenon was proposed to be the main cause of embrittlement in epoxies since sub-glassy mobility plays a key role in the plasticity of glassy polymers [14]. It is, unfortunately, tricky to predict the residual sub-glassy mobility (expressed by the area under the peak of β relaxation) using a kinetic model.

The present paper, therefore, aims at identifying the simple structure–properties relationships involved in embrittlement so as to « link » the output data of kinetics models (oxygen absorption and concentration in oxidation products) with mechanical properties (ultimate stress and strain, and toughness and the elastic modulus) of several oxidized epoxy amine networks. This will be helpful for a direct prediction of failure in the case of thin films (typically epoxy coatings). In the case of the prediction of the residual mechanical properties of “thick” samples (typically where oxidation is governed by oxygen diffusion), a simulation approach was previously used [15]. The inputs were “local” mechanical properties, varying with the oxidation level. Since kinetic models can describe the change of oxidation as a function of time and polymer thickness, using relationships between oxidation level (for example, concentration in oxidation product) and mechanical parameters is expected to simplify the prediction of failure for a bulky material.

## 2. Experimental Procedure

### 2.1. Materials

**Figure 1:**
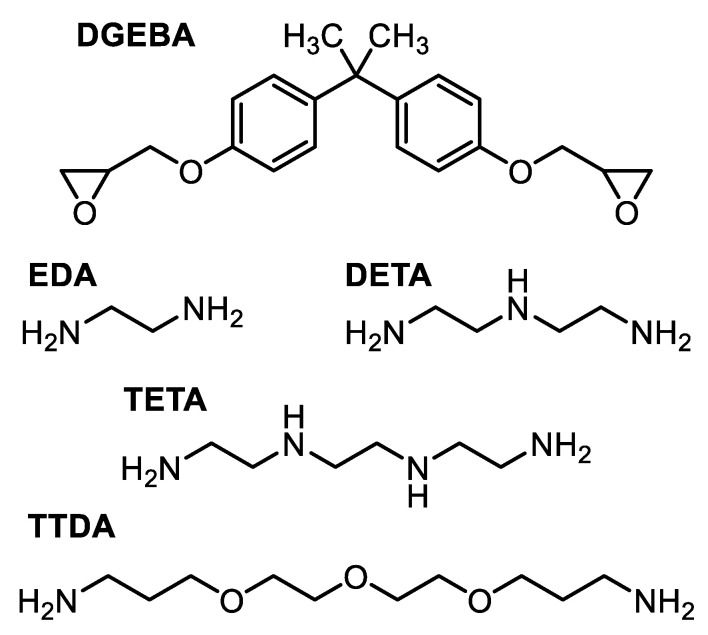
Chemical formulas of epoxide prepolymer and hardeners used to synthesize the networks under investigation.

Several epoxy-amine thermoset systems composed from the same epoxide prepolymer DGEBA were used for this study of their macromolecular properties. D.E.R. 332 (CAS 1675-54-3, with EEW 171–175 g/eq) was chosen for this purpose as it consists of almost only mono-units of DGEBA, making the length of chains between crosslinks more predictable. Networks were cured with three different amine hardeners: ethylene diamine (EDA, CAS 107-15-3), diethylenetriamine (DETA, CAS 11-40-0, with AHEW 20.6 g/eq) and 4,7,10-trioxa-1,13-tridecanediamine (TTDA, CAS 4246-51-9, with AHEW 55.1 g/eq). All these chemical products (with formulas developed in Figure 1) were provided by Sigma-Aldrich. 

A commercial epoxy resin was also studied. It was obtained by mixing a type of DGEBA prepolymer together with a mixture containing triethylenetetramine (TETA, CAS 112-24-3) and a cycloaliphatic diamine hardener. This industrial epoxy system also contained benzylic alcohol, which is used as a plasticizer to improve the processing and the curing [16] in practical conditions typically encountered in the field of civil engineering.

Systems were cured according to the following procedures:

- DGEBA-TTDA was cured for 1 h at 60 °C with a heating press and post-cured for 3 h in a vacuum at 80 °C.
- DGEBA-EDA and DGEBA-DETA were cured according to the following experimental procedure: After mixing the prepolymers in stoichiometric proportions (according to the equivalent weights previously listed), the blends were set to thicken for up to 3 h in closed vials at ambient room temperature (actual time being heavily dependent on the nature and volume of the mixture and atmospheric conditions). Once they were thick enough, the blends were pressed using a hydropress for 1 h at 50 °C and 1 h at 90 °C. They were then post-cured for 30 min at 170 °C under vacuum.
- Commercial epoxy was cured at room temperature (48 h).

**Figure 2:**
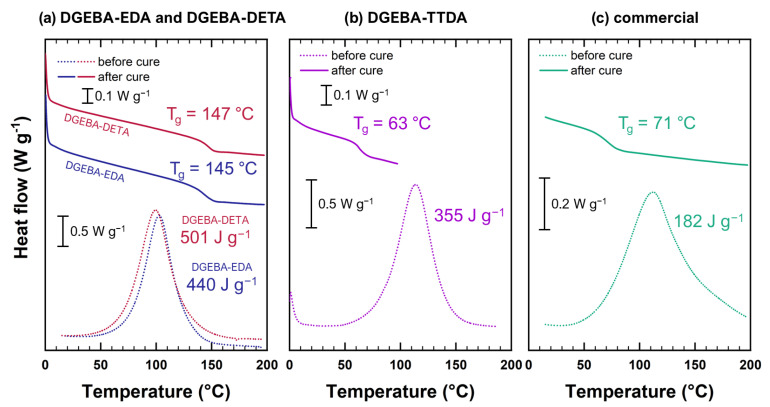
DSC thermograms before and after curing for DGEBA-EDA and DGEBA-DETA (**a**), DGEBA-TTDA (**b**) and commercial epoxy (**c**) under investigation.

Thermograms for uncured and fully cured networks are given in Figure 2. Curing parameters are recalled in Table 1.

No exothermic peak was detected using Differential Scanning Calorimetry (DSC) for unaged samples, and the curing process was therefore assumed to be complete. It was carried out using a DSC Q10 (TA Instruments©, New Castle, DE, USA) at 10 °C min^−1^ with samples of about 15 mg. The same machine was later used to follow glass transition temperatures of aged samples. The heating rate was chosen in line with literature [17,18].

### 2.2. Exposure Conditions

Punched samples were aged at 80, 120, 160 and 200 °C in ventilated ovens (AP60, System Climatic Service, Saint Ouen L’Aumone, France).

## 3. Characterization

### 3.1. Fourier Transform InfraRed Spectroscopy

FTIR spectroscopy in transmission mode was carried out using a Frontier spectrophotometer (PerkinElmer) by averaging eight scans with a resolution set at 4 cm^−1^ between 400 cm^−1^ and 4000 cm^−1^!

### 3.2. Differential Scanning Calorimetry

In order to measure the glass transition of unaged and aged samples, the latter were submitted to the following thermal cycle: after equilibrating at 100 °C to clear structural ageing relaxation, they were quickly equilibrated at 0 °C and then heated to 150 °C at 10 °C min^−1^ during the heating ramp. Analyses were done using a DSC Q10 apparatus (TA Instruments) with aluminium pans and lids under 50 mL min^−1^ nitrogen flow. Results were analysed using TA Analysis software.

### 3.3. Mechanical Test

**Figure 3:**
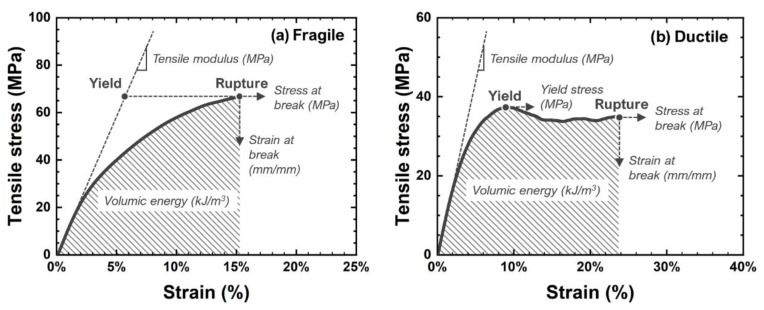
Exploitation of stress-strain curves in the case of fragile epoxies (**a**) and ductile epoxies (**b**).

H4 type samples were punched before ageing and tested using an Instron 4301 tensile apparatus equipped with a 100 N force sensor. Tests were carried out at a 1 mm min^−1^ rate in tensile mode. Data were extracted and are illustrated in Figure 3.

## 4. Results and Discussion

### 4.1. Accumulation of Stable Oxidation Products

**Figure 4:**
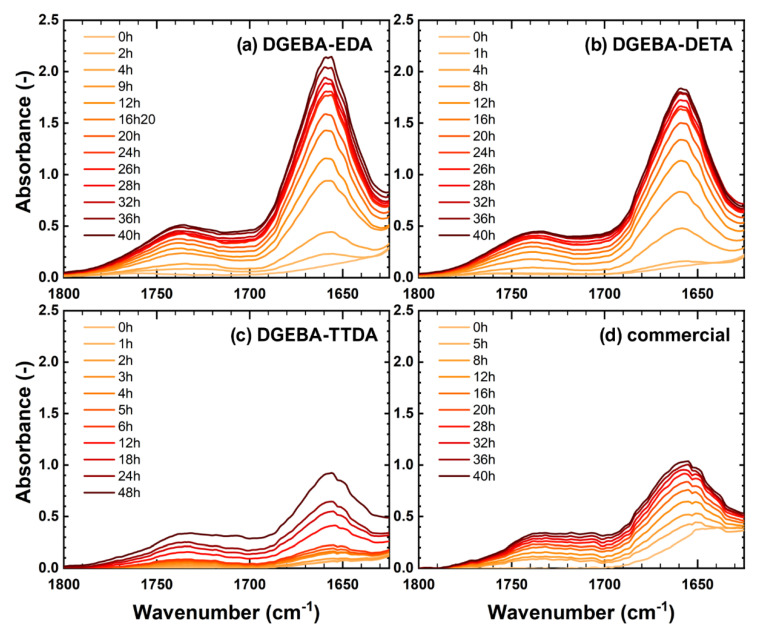
FTIR spectra in the carbonyl region for DGEBA-EDA (**a**), DGEBA-DETA (**b**), DGEBA-TTDA (**c**) and commercial epoxy (**d**) aged at 160 °C in ventilated ovens.

Thermal oxidation resulted in the appearance of stable products, which is presented in Figure 4. They depict the major appearance of amides (characterized by a peak centred at about 1660 cm^−1^) [7,10].

The band centred at 1730 cm^−1^ stems from several species, resulting from the oxidation of several groups located either on DGEBA groups or on the hardeners. In the case of DGEBA-TTDA, it was proposed that the oxidation of ethylene glycol moieties located on the hardener side results in esters [19]. In each case, the product absorbing at 1660 cm^−1^ predominated. This is ascribed to the amide formation resulting from the oxidation of methylene in the vicinity of nitrogen atoms (being the crosslink nodes). In the case of DGEBA-EDA and DGEBA-DETA, the methylene held by hardeners groups would be more sensitive to oxidation than those held by DGEBA groups, as discussed in another work [20]. 

### 4.2. Changes in Mechanical Properties

**Figure 5:**
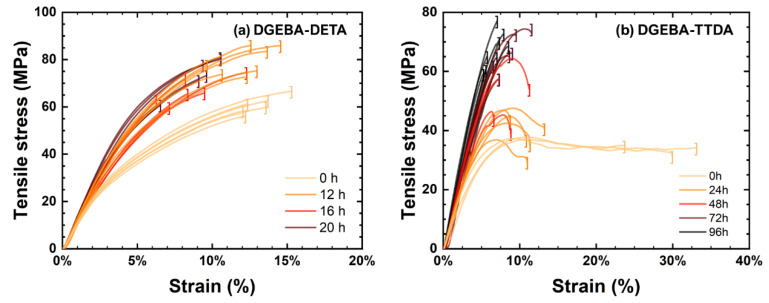
Typical stress-strain curves before and after several ageing durations at 160 °C for DGEBA-DETA (**a**) and DGEBA-TTDA (**b**).

**Figure 6:**
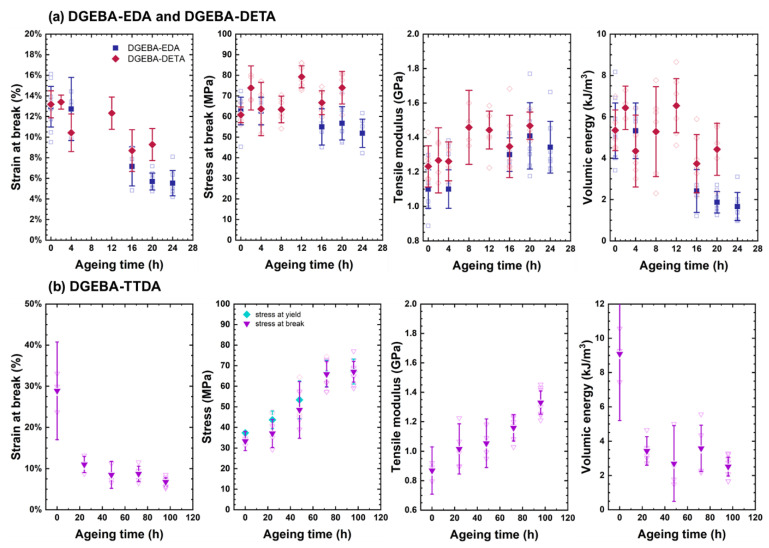

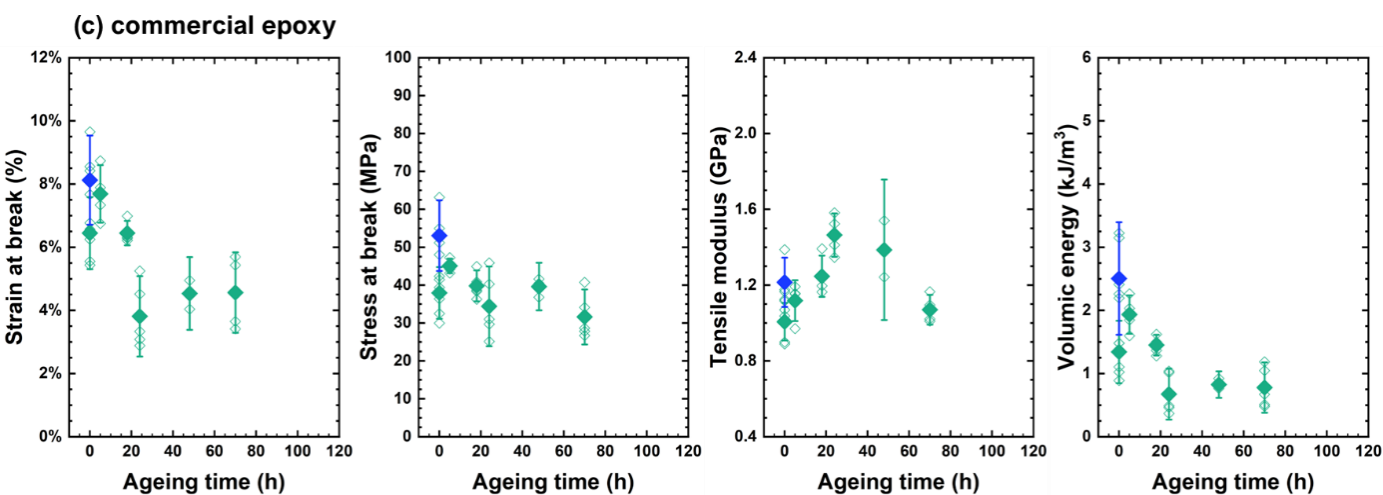
Changes in mechanical properties vs. time for DGEBA-EDA and DGEBA-DETA (**a**), DGEBA-TTDA (**b**) and commercial epoxy (**c**).

A typical comparison of stress-strain curves for unaged and aged systems is illustrated in Figure 5. During oxidation, mechanical properties changed, as depicted in Figure 6. 

They can be summarized as follows:

➀ The elastic modulus continuously increased.
➁ Ultimate strain continuously decreased.
➂ Stress at break first increased in link with the increase in the elastic modulus and decreased in link with the decrease in ultimate strain. Its changes were driven by two opposite trends: (i) the total loss of plasticity, so that the decrease in ultimate stress is linked to the decrease in ultimate strain, and (ii) the «antiplasticization», due to loss of plasticizer and loss of beta relaxation inducing the increase in ultimate stress. In DGEBA-TTDA, another phenomenon must be taken into account: the increase in yield stress due to crosslinking, which seems to have induced an increase in ultimate stress (stress at break). Lastly, the comparison of DGEBA-EDA, DGEBA-DETA and commercial epoxy suggests that plasticizer loss (if any) had a negligible influence. As a matter of fact, the stress-strain curves for commercial epoxy before and after evaporation under vacuum of plasticizer are depicted in Appendix A and do not show significant differences.
➃ The volumic energy (determined as the “area under the stress-strain curve”) decreased with the decrease in ultimate strain (both phenomena being undoubtedly related).

There were no significant differences between DGEBA-EDA and DGEBA-DETA, presumably because both samples display very close T_g_, molecular structure and reactivity [20]. Despite a much lower T_g_, the industrial epoxy and DGEBA-TTDA also followed the same behaviour, apart from ultimate stress in the case of DGEBA-TTDA. This will be commented on in the next section.

### 4.3. Correlation between Chemical and Mechanical Changes

The last step was to establish a relationship between the main stable tracker for chemical changes (the formation of amides) and the depletion of mechanical properties, similarly to results for polypropylene where the FTIR band for methyl groups (1456 cm^−1^) was shown to be correlated with changes in crystallinity, molar mass and micro-hardness [21]. 

**Figure 7:**
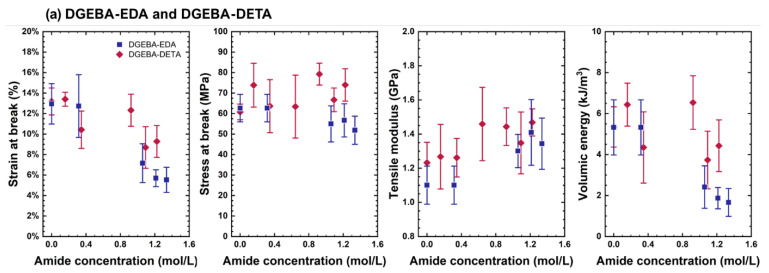

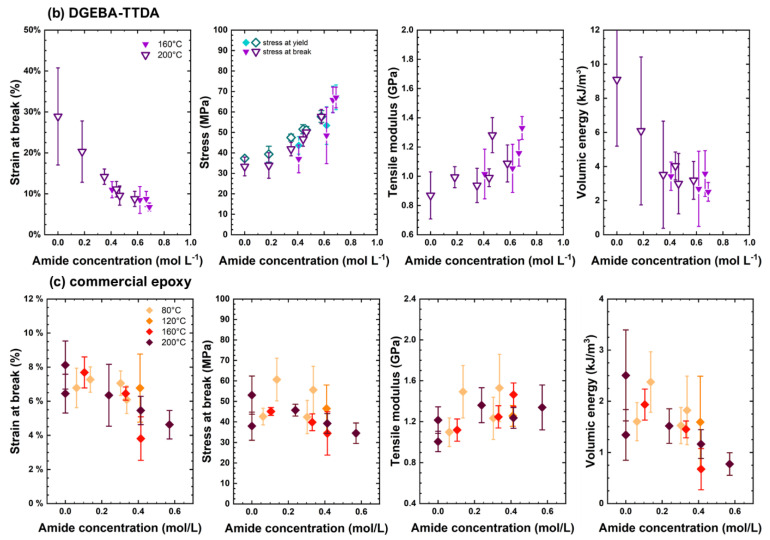
Structure properties involved in the degradation induced embrittlement of DGEBA-TTDA (**a**), DGEBA-EDA and DGEBA-DETA (**b**) and industrial epoxies (**c**).

For that purpose, absorbances due to amides were first converted into concentrations with the Beer–Lambert Law, using a molar absorptivity value equal to 400 L mol^−1^ cm^−1^ [22]. Those concentration values were then correlated with mechanical properties, as depicted in Figure 7, showing several trends.

Notably, the elastic modulus increased with the concentration of amides. The ratio was about 0.2–0.3 GPa L mol^−1^ for model systems and higher in the commercial epoxy. This result seems at first sight counterintuitive since DGEBA-DETA and DGEBA-EDA undergo mainly chain scission [23], whereas DGEBA-TTDA [24] and the commercial epoxy resin mainly undergo crosslinking [25]. However, the main reason for this is the following: The elastic modulus of thermoset polymers (in a glassy state) is given by [13,14]:

(1) $$E\left(T\right)=E_{0}\cdot \left(1-\alpha \frac{T}{T_{g}}\right)-\sum_{i}∆E_{i}$$

where *E*_0_ depends on the cohesive energy [26], α expresses the thermal expansion effect and Δ*E_i_* corresponds to the drop corresponding to the activation of a sub-glassy mobility. In the case of epoxies, the main relaxation is β relaxation, so that between *T_g_* and *T_β_*:

(2) $$E\left(T\right)=E_{0}\cdot \left(1-\alpha \frac{T}{T_{g}}\right)-∆E_{\beta }$$

Using the orders of magnitude reported for glassy polymers, it is easy to verify that the first term on the right-hand side of Equation (2) is negligible compared to the second one. In other words, *T_g_* changes (induced either by chain scission or by crosslinking) have a minor effect on the elastic modulus compared to the effect of mobility on ΔE_β_ (about 1 GPa). In other words, the consumption of small groups responsible for sub-glassy mobility is the main explanation for an increase in the modulus. To go further, let us assume that the amplitude of β relaxation is directly proportional to the (residual) concentration of hydroxypropylether groups (denoted by c), which can easily be deduced from the structure of the constitutive repetitive unit:

(3) $$∆E_{\beta }=∆E_{\beta 0}\cdot \frac{c}{c_{0}}=∆E_{\beta 0}\cdot \frac{c_{0}-\left[Amide\right]}{c_{0}}$$

According to Table 2, one sees that ΔEβ_0_/c_0_ actually is on the order of 1 GPa, which is a reasonable order of magnitude. In the case of the commercial epoxy, the slope of the elastic modulus vs. amide is higher, but this can be easily explained by plasticizer loss occurring during ageing.

Despite its first increases due to the antiplasticization effect reported above, the changes in ultimate stress seem in greater part explained by the progressive decrease in ultimate strain. In the specific case of DGEBA-TTDA, a yield stress was observed. It increased regularly during ageing. This trend is straightforward to discuss since yield stress is linked to glass transition according to Kambour’s law [27]:

σ_Y_ = C(T_g_ – T) + σ_y0_ (4)

**Figure 8:**
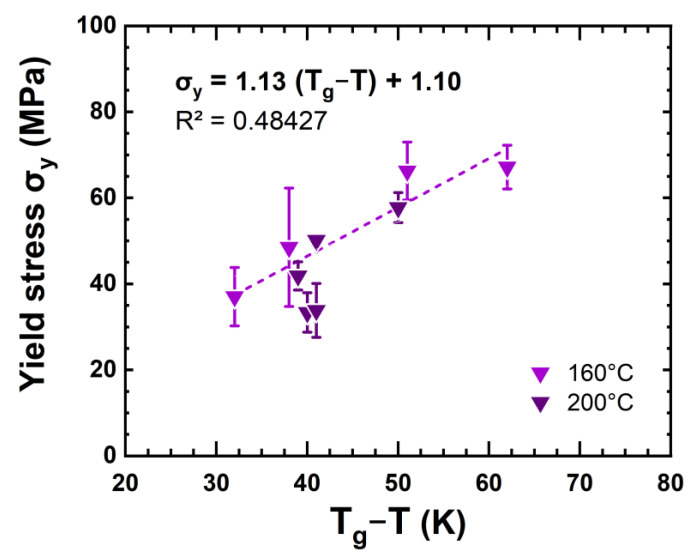
Stress at yield vs. glass transition and carbonyl concentration for DGEBA-TTDA.

This result is depicted in Figure 8. An approximated engineering rule is obtained in line with comparable equations found in the literature (in particular with a slope of C~1) [28,29]. However, it is difficult at this stage to establish a direct link with amide concentration since ageing can lead to chain scission or crosslinking, depending on the flexibility of segments [24].

Another important trend was that the ultimate strain and volumic energy decreased almost linearly with amide content. The effect of oxidation seems higher for DGEBA-TTDA than for other networks (for example, by inspecting the slope of volumic energy vs. amide concentration). However, it is worth highlighting that a drop of 50% of initial elongation at break was observed only at a high concentration of amides (typically more than 0.25 mol L^−1^ for DGEBA-TTDA and up to 1 mol L^−1^ for DGEBA-EDA and DGEBA-DETA) contrary to polyolefins, for example, where embrittlement is obtained at a relatively low degree of oxidation (typically [carbonyls] < 0.1 mol L^−1^) [30,31].

Studies from the literature data have shown comparable behaviour: For example, DGEBA-IPDA becomes brittle for [amide] ~0.6 mol L^−1^, whereas c_0_ ~4.7 mol L^−1^ [19]. This means that a perfect understanding of chemistry up to high conversion levels is needed to establish a reliable kinetic model for predicting embrittlement. From an applied point of view, the value of this “critical” amide concentration can be commented on regarding the initial hydroxypropylether concentration: it corresponds to about 10% of its initial concentration.

According to Figure 7c, data obtained for the commercial epoxy at several temperatures from 80 to 200 °C overlap reasonably. This suggests that the embrittlement induced by oxidation can be observed at enhanced temperatures usual for accelerated ageing and extrapolated to service temperatures. This also means that the development of a kinetic model predicting chemical changes in accelerated conditions can be used for predicting the lifetime of epoxy parts in natural ageing.

## 5. Conclusions

Model epoxy-amine networks consisting of DGEBA cured with trioxa-tridecanediamine, or ethylene diamine, or diethylene triamine, or commercial system consisting triethylenetetramine and a cycloaliphatic polyamine hardener was cured. They were submitted to thermal ageing at various temperatures (from 80 to 200 °C). At the molecular scale, and despite differences in chemical structure, all networks were shown to undergo fast oxidation (occurring in less than 24 h at 160 °C) with the predominant formation of amides as stable chemical trackers. At the macroscopic scale, mechanical properties were followed by classical stress-strain curves in tensile mode. The elastic modulus increased mainly to the previously documented effect of antiplasticization associated with the destruction of small groups responsible for sub-glassy mobility. Ultimate strain and volumic energy for failure (i.e., area under stress-strain curves) progressively decreased. A correlation between chemical trackers and mechanical parameters was observed for all systems under investigation. Building a kinetic model for the prediction of degradation in service conditions is possible by extrapolating data obtained by accelerated ageing at high temperature. 

## Figures and Tables

**Table 1:** Curing parameters of epoxy systems.

System	$T_{\mathrm{onset}}$ (°C)	$T_{\max}$ (°C)	ΔH (J g^−1^)	$T_{g}^{\mathrm{final}}$ (°C)
DGEBA-TTDA	82	114	355	63
DGEBA-EDA	76	103	440	145
DGEBA-DETA	70	100	501	147
Commercial epoxy	66	112	182	71

**Table 2:** Description of repetitive units in model epoxy-amine systems.

System	M_UCR_ (g mol^−1^)	Number of Hydroxypropylether Groups/UCR	c_0_ (mol L^−1^)
DGEBA-TTDA	904	4	~5.3
DGEBA-EDA	744	4	~6.8
DGEBA-DETA	904	5	~6.6

**Figure A1:**
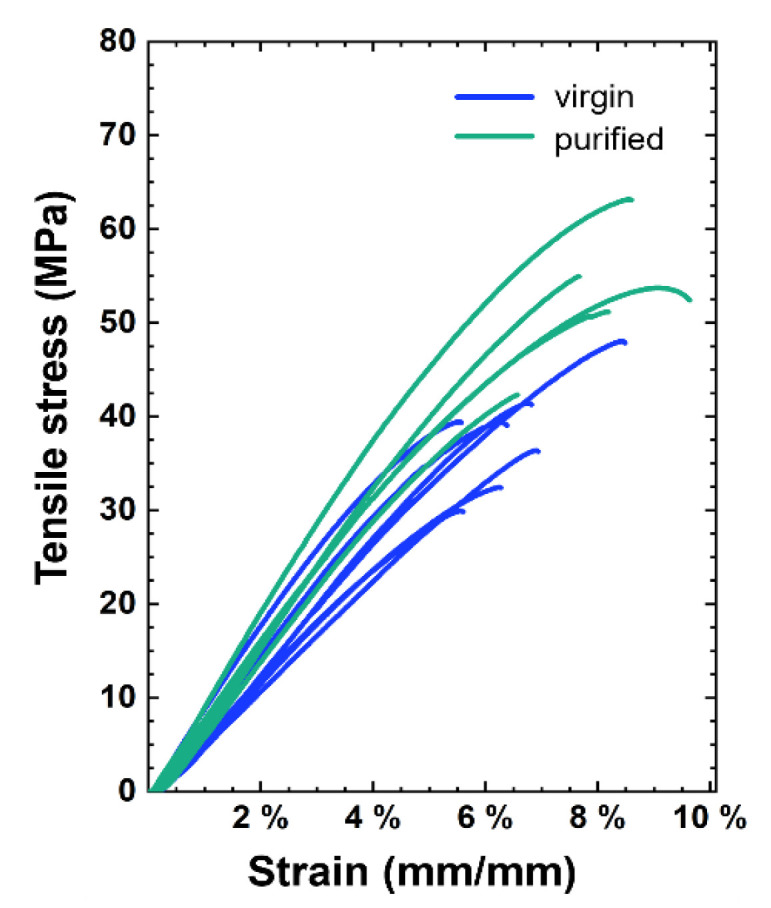
Effect of plasticizer loss on mechanical properties for commercial epoxy.

## Data Availability

Data are available on request.
